# Same old song and dance: an exploratory study of portrayal of physical activity in television programmes aimed at young adolescents

**DOI:** 10.1186/s13104-018-3554-8

**Published:** 2018-07-11

**Authors:** Heather O’Reilly-Duff, Paul Best, Mark A. Tully

**Affiliations:** 1 Centre for Public Health, School of Medicine, Dentistry and Biomedical Sciences, Queen’s University Belfast, Belfast, Northern Ireland, UK; 2 Centre for Evidence and Social Innovation, School of Social Sciences, Education and Social Work, Queen’s University Belfast, Belfast, Northern Ireland, UK; 3 UKCRC Centre of Excellence for Public Health Research (Northern Ireland), Queen’s University Belfast, Belfast, Northern Ireland, UK; 4 Institute of Mental Health Sciences, School of Health Sciences, Ulster University, Newtownabbey, Northern Ireland, UK

**Keywords:** Physical activity, Exercise, Adolescent, Television

## Abstract

**Objective:**

Exposure to health-related behaviours on television has been shown to influence smoking and drinking in young people, but little research has been conducted on the portrayal physical activity. The aim of the current project was to explore the portrayal of physical activity in television programmes aimed specifically at adolescent females. Content analysis of 120 episodes of four popular adolescent television programmes was performed. Information on the type and context of physical activity, motivating factors and characters involved was recorded.

**Results:**

Physical activity was portrayed 122 times, for a duration of 1 h and 31 min (3.2% of total viewing time). Physical activity was mainly portrayed as part of an informal activity as part of a group activity. Over half (53.2%) of scenes portrayed activity been carried out by teenagers. The types of activities portrayed were mostly of vigorous intensity (76.2%), for recreational purposes (78.7%) such as dancing (54.1%) and running (11.5%), and motivated by enjoyment. This study highlights that physical activity is portrayed infrequently, and often with a skewed representation of type of activity. There may be an opportunity to influence physical activity in young adolescents through the positioning of positive images of an active lifestyle in the media.

**Electronic supplementary material:**

The online version of this article (10.1186/s13104-018-3554-8) contains supplementary material, which is available to authorized users.

## Introduction

Physical inactivity is a major cause of chronic conditions, such as heart disease, cancer, obesity, stroke, type 2 diabetes and mental health problems [1]. A substantive decline in physical activity occurs during early adolescents, with only 38% of adolescent females meeting the recommended amount of physical activity per day [2], making this an important group to research.

### Media influences on physical activity levels

The media has on influential role on perceived social norms and attitudes to physical activity [3]. Some behavioural theories also emphasise the significant influence other people, including role models, exert on the likelihood to engage in physical activity. Bandura’s social learning theory [4] proposes that behaviour is learnt through observation. Therefore, models of behaviour viewed on television may provide young people with examples of physical activity to imitate or may reinforce, or challenge, beliefs regarding physical activity.

Previous research has documented how the portrayal of various health-related behaviours can influence the lifestyles of adolescent viewers. The frequency of alcohol and tobacco imagery and marketing within television programs and films, have been shown to influence smoking rates and alcohol consumption [5]. It is estimated that exposure to smoking on television will influence 6.4 million children to try smoking, of which 90% will begin smoking before they reach the age of 18 [6].

Only three previous studies have documented the portrayal of physical activity on television. Simpson et al. [7] examined three randomly selected episodes from of the five most popular Disney channel television programmes. Exercise was depicted in 53% of the 15 episodes analysed. Of this, 21% was resistance to exercise and 13% portrayed exercise as a positive behaviour. Portrayal of exercise was not the main focus of this study and therefore very little detail is provided. Scully et al. [8] analysed the content of television programmes aimed at young children in the UK and Ireland over a 5-day period. They showed that 7.7% of screen time depicted exercise or physical activity. Interestingly male characters were more frequently depicted taking part in exercise than girls of the same age. Finally, Gietzan et al. [9] analysed the physical activity content of 75 episodes from 25 different shows selected by a cohort of young people aged 12–18 years (both males and females). They demonstrated that physical activity was portrayed five times in every episode, with males portrayed doing activity more often than females. Further research is required to describe in more detail the portrayal of physical activity in television programmes aimed at younger adolescents. Early adolescence is an important age group to research, as physical activity levels decline rapidly and personal lifestyle choices and behaviour patterns are established [10].

The aim of the current project was therefore to conduct a quantitative content analysis to explore the portrayal of physical activity in television programmes aimed specifically at young adolescents.

## Main text

### Methods

A quantitative content analysis was conducted on four popular adolescent television programmes. Content analysis is a research technique for the objective, systematic and quantitative description of communication [11].

#### Choice of programmes and episodes

The programmes included in this study (*iCarly*, *Victorious, Wizards of Waverly Place* and *Jessie*) were chosen because they primarily target younger adolescents aged 10–14 years (known as ‘*tweens*’), have global appeal, are well established, have won prestigious awards for popularity and were available to researchers via online streaming services. The main characters in these shows are mainly teenagers, but also include some children and young adults. All have reported viewing figures between 1 and 11 million people [12] when initially broadcasted in the USA, and have had similar popularity in the UK and beyond. *iCarly* (broadcast 2007–2012) and *Victorious* (broadcast 2010–2012) were distributed by Nickelodeon and *Wizards of Waverly Place* (broadcast 2007–2013) and *Jessie* (broadcast 2011–2015) were distributed by the Disney Channel.

#### Data recording

Thirty episodes (Additional file 1) from each programme were chosen at random, using an online tool (https://www.randomizer.org). Initially, ten episodes were independently analysed by two researchers to test the data recording process. The reliability was assessed using intra-class correlations (ICCs). The remainder of the programs were analysed by one investigator, who had been involved in the initial trial observation. Each programme was watched until an occurrence of physical activity was observed. Initially the instance was watched until the physical activity had ceased. The programme was then paused and the characteristics recorded. The scene was then replayed to check details and record the length of the occurrence.

#### Data recording instrument

A coding instrument (Additional file 2) was developed to record the portrayal of physical activity, based on the method described by Skully et al. [8]. An occurrence of physical activity was defined as visual, verbal (i.e. when the characters are discussing physical activity) or visual-verbal reference to any form of physical activity being undertaken in a scene.

In each scene, any physical activity that took place as the main event or in the foreground or background was recorded. For each physical activity occurrence, the duration (seconds), intensity (mild, moderate, vigorous activity), character age (child, teen, adult or mixed), sex, and motives were recorded. The compendium of physical activities [13] was used to assess the intensity of activities according to their metabolic equivalent (MET). These were then classified as mild (< 3 METS intensity, moderate (3–6 METs) or vigorous (> 6 METs) intensity.

Formal physical activity was recognized as activity that took place under instruction, a competition (e.g. football match, talent show or activity included in a performance e.g. activity in a play, singing and dancing on stage). A physical activity was classed as informal if it did not fulfil the above requirements. Individual occurrences were classified when only one individual was involved, whereas group/team when more than one person was involved.

The domain of the physical activity was recorded to determine the nature in of the activity (e.g. occupational—as required by work or school, recreational—activity undertaken in leisure time, active travel—activity as a means of transport). Motivations were recorded to give insight as to why the physical activity was being undertaken (e.g. for enjoyment, as part of a performance, for fitness or as part of daily routine). The sentiment that resulted as an outcome of the physical activity (positive, negative or neutral) was also recorded. This was noted from either the body language of the character or their spoken word. Examples of positive outcomes included celebrating winning, enjoyment or benefits gained.

#### Statistical analysis

Data analysis was conducted using SPSS version 23 (IBM Corp, USA). Descriptive statistics for categorical variables included overall number, duration (seconds) mean and standard deviation and frequency (%) of occurrences per episode. Chi squared tests were used to assess differences in the portrayal of physical activity between Nickelodeon and Disney channel programmes. As the study did not involve human participants, ethical approval was not required.

### Results

A total of 120 episodes were analysed, equating to a viewing time of 46 h and 45 min. The level of agreement between observers, assessed using ICCs, was 1 (indicating 100% agreement) for all categories recorded apart from sentiment (ICC = 0.76). Physical activity was portrayed on screen 122 times, for a duration of 1 h and 31 min (3.2% of total viewing time). However, 59/122 (47.6%) episodes showed no occurrences of physical activity (Table 1).

**Table 1:** Characteristics of physical activity portrayal in television programmes aimed at adolescent girls

	Total (n = 122)	Disney channel (n = 52)	Nickelodeon (n = 70)	Difference between channels *p*-value
Portrayal				0.001
Visual	68 (55.7%)	21 (40.4%)	47 (67.1%)	
Verbal	18 (14.8%)	6 (11.5%)	12 (17.1%)	
Visual + verbal	36 (29.5%)	25 (48.1%)	11 (15.8%)	
Focus within the scene				0.005
Main event	38 (31.1%)	8 (15.4%)	30 (42.9%)	
Foreground	62 (50.8%)	32 (61.5%)	30 (42.9%)	
Background	22 (18.1%)	12 (23.1%)	10 (14.2%)	
Context				0.27
Group formal	14 (11.5%)	4 (7.7%)	10 (14.2%)	
Group informal	74 (60.7%)	29 (55.8%)	45 (64.3%)	
Individual formal	5 (4.1%)	3 (5.7%)	2 (2.9%)	
Individual informal	29 (23.7%)	16 (30.8%)	13 (18.6%)	
Sex				0.78
Male	29 (23.8%)	14 (26.9%)	15 (21.4%)	
Female	30 (24.6%)	12 (23.1%)	18 (25.7%)	
Mixed	63 (51.6%)	26 (50%)	37 (52.9%)	
Age				0.001
Child	9 (7.4%)	9 (17.3%)	0 (0%)	
Teen	65 (53.3%)	20 (38.5%)	45 (64.2%)	
Adult	10 (8.2%)	5 (9.6%)	5 (7.1%)	
Mixed	38 (31.1%)	18 (34.6%)	20 (28.7%)	
Motivation for taking part				0.08
Enjoyment	73 (59.8%)	33 (63.5%)	40 (57.1%)	
Functional	9 (7.4%)	2 (3.8%)	7 (10%)	
Gain fitness	10 (8.2%)	8 (15.4%)	2 (2.9%)	
Gain muscle	1 (0.8%)	0 (0%)	1 (1.3%)	
Other	3 (2.5%)	0 (0%)	3 (4.3%)	
Performance	12 (9.8%)	3 (5.8%)	9 (12.9%)	
School curriculum	13 (10.7%)	6 (11.5%)	7 (10%)	
Social	1 (0.8%)	0 (0%)	1 (1.4%)	
Sentiment				0.002
Positive	45 (36.9%)	10 (19.3%)	35 (50%)	
Negative	27 (22.1%)	14 (26.9%)	13 (18.6%)	
Neutral	50 (41%)	28 (53.8%)	22 (31.4%)	

#### General characteristics

Physical activity was mainly portrayed as part of an informal activity as part of a group activity (60.7% of occurrences) (Table 1). A similar proportion of scenes portrayed only males (23.8%) or only females (24.6%) taking part in physical activity, but most scenes portrayed a mix of males and females (51.6%). Not surprisingly given the nature of the programmes, over half (53.2%) of scenes portrayed activity been carried out by teenagers (Table 1).

Of note, the types of activities portrayed were mostly vigorous intensity (76.2%), for recreational purposes (78.7%), such as dancing (54.1%), running (11.5%) and other (11.3%) which included activities such as water fights and playing table tennis (Table 2). Dancing was the most common activity, which was portrayed in an informal setting as part of a group (70% of scenes involving dance were as part of an informal group). The motivation for taking part in physical activity appeared to be for enjoyment (59.8%) and the expressed outcome was neutral (41% of occurrences), positive (36.9%) or negative (21.8%) (Table 2). Positive outcomes were commonly displayed by linking physical activity to positive emotions. For example, in *Victorious*, which is set in a preforming arts school, many of their dance performances received positive responses i.e. cheering, and clapping resulting in happiness and pride in the characters involved. An example of negative outcomes was when a character had negative feelings towards participating in a boxing match, due to fear of losing and getting hurt (*iCarly* season 2 episode 21).

**Table 2:** Intensity and type of physical activity portrayed in television programmes aimed at adolescent girls

	Total (n = 122)	Disney Channel (n = 52)	Nickelodeon (n = 70)	Difference between channels *p*-value
Intensity				0.10
Mild	8 (6.5%)	1 (1.9%)	7 (10%)	
Moderate	21 (17.2%)	7 (13.5%)	14 (20%)	
Vigorous	93 (76.2%)	44 (84.6%)	49 (70%)	
Physical activity domain				0.53
Active travel	6 (4.9%)	4 (7.7%)	2 (2.9%)	
Domestic	3 (2.4%)	1 (1.9%)	2 (2.9%)	
Occupational	16 (13.1%)	5 (9.6%)	11 (15.7%)	
Recreational	96 (78.7%)	42 (80.8%)	54 (77.1%)	
Other	1 (0.8%)	0 (0%)	1 (1.4%)	
Type of physical activity				0.45
American football	1 (0.8%)	1 (1.9%)	0 (0%)	
Basketball	1 (0.8%)	1 (1.9%)	0 (0%)	
Boxing	3 (2.5%)	0 (0%)	3 (4.3%)	
Dancing	66 (54.1%)	27 (51.9%)	39 (55.7%)	
Fighting	4 (3.3%)	0 (0%)	4 (5.7%)	
Golf	1 (0.8%)	0 (0%)	1 (1.4%)	
Gymnastics	2 (1.6%)	1 (1.9%)	1 (1.4%)	
House cleaning	3 (2.5%)	1 (1.9%)	2 (2.9%)	
Playing games	1 (0.8%)	1 (1.9%)	0 (0%)	
Running	14 (11.5%)	7 (13.5%)	7 (10%)	
Surfing	1 (0.8%)	0 (0%)	1 (1.4%)	
Swimming	1 (0.8%)	0 (0%)	1 (1.4%)	
Tennis	3 (2.5%)	1 (1.9%)	2 (2.9%)	
Walking (brisk)	1 (0.8%)	0 (0%)	1 (1.4%)	
Weight lifting	1 (0.8%)	0 (0%)	1 (1.4%)	
Working out	5 (4.1%)	3 (5.8%)	2 (2.9%)	
Other	14 (11.5%)	8 (15.4%)	6 (8.6%)	

#### Comparison of channels

Overall, the two Nickelodeon channel programmes portrayed more occurrences of physical activity compared to the two Disney channel programmes (70 vs 52) (Table 1). There was a significant difference between channels in the age groups taking part in activity (p = 0.001; Table 1). Nickelodeon programmes showed no children taking part in physical activity, but portrayed mostly teens (64.2%). The Disney channel programmes associated significantly more occurrences of physical activity with positive outcomes (p = 0.002; Table 1).

### Discussion

To our knowledge, this is the first study to analyse the portrayal of physical activity in television programmes specifically aimed at young adolescents. This study has demonstrated that physical activity is portrayed infrequently (just half of episodes), and considerably less frequently than previous studies have shown in programmes aimed at broader audiences [9]. Physical activity was portrayed in positive light, involving recreational activity, the most common of which was dancing, which was twice as frequently portrayed in the programmes analysed as any other activity. Scully et al. [8] also demonstrated dancing is the most prevalent activity in programmes aimed at young children. There are many potential mental and physical health benefits that arise from encouraging young adolescents to engage in dance [14], in addition to the contribution dance classes makes to the overall level of physical activity in young adolescents [15]. Participation of young adolescents in dancing has increased in recent years [16], but it is still not the most common type of physical activity in adolescents, who are more likely to engage in activities such as cycling, swimming, and ball sports [17]. Biased portrayals on television may influence choice of physical activity by cultivating biased perceptions of reality [5]. Though this has not been investigated for physical activity, it has been shown that viewing smoking on television influences earlier onset of smoking initiation in adolescents [18]. Maibach [3] notes that portraying physical activity as uncomfortable or unpleasant may serve to influence viewers to develop a negative biased perception of physical activity. This current project has shown that activity is generally portrayed in a positive light, which should reinforce the enjoyable aspects of been active.

### Conclusion

This study has demonstrated that physical activity is portrayed infrequently, sporadically and with a skewed representation of type of activity shown, with dance being the predominant form. More research is needed to fully understand the relationship between the physical activity portrayed on television and its effect on the physical activity of viewers, but the portrayal may have an important contribution in terms of cultivating and framing of physical activity in young adolescents.

## Limitations

There are many strengths to this analysis. The channels chosen are the most popular worldwide for this age group and gender, and their content reaches a large number of people. A greater number of episodes per programme have been included in this study, than previous research. The study draws attention to the amount of further research needed to understand the influence that media and television has on adolescent females.

There are a number of limitations that should be noted. Only one person coded the occurrence of physical activity in this study in all programmes, which may have led to a biased coding. However, in the initial test of the data recording process, agreement between independent observers was high. Only two television programmes were analysed from each channel, therefore the findings and evaluation are limited to the portrayal in these specific programmes. Had more programmes been included, it may have yielded different findings. Episodes were randomly selected for viewing in an attempt to negate any influence changing story lines across multiple seasons. In addition, one of the programmes (Victorious) was set in a performing arts school, which may bias the study results.

## Additional files

**Additional file 1.** Episodes randomly selected for analysis.

**Additional file 2.** Data Recording Form.

## Abbreviations

ICCs: intra-class correlations
MET: metabolic equivalent
