# Supplementary material for: Same old song and dance: an exploratory study of portrayal of physical activity in television programmes aimed at young adolescents
Source: BMC Res Notes. 2018 Jul 11;11:458. doi: 10.1186/s13104-018-3554-8 (PMC6042342; doi:10.1186/s13104-018-3554-8)
Supplement: Supplementary file 1 — Additional file 1. Episodes randomly selected for analysis. [file 13104_2018_3554_MOESM1_ESM.docx]

**Additional file 1: Episodes randomly selected for analysis**

**iCarly**

Episodes 6, 13, 14, 16, 20, 29, 30, 32, 34, 40, 45, 46, 58, 60, 61, 69, 71, 72, 78, 80, 81, 82, 83, 84, 94, 95, 98, 104, 107, 109

**Victorious**

Episodes 1, 3, 4, 5, 8, 9, 10, 11, 12, 13, 14, 15, 16, 17, 19, 24, 25, 28, 29, 31, 32, 33, 34, 39, 40, 41, 44, 45, 49, 51

**WOWP**

Episodes 4, 5, 17, 20, 24, 29, 31, 35, 36, 50, 52, 54, 57, 58, 62, 68, 70, 72, 80, 85, 86, 89, 92, 94, 95, 100, 101, 102, 104, 105

**Jessie**

Episodes 2, 3, 6, 8, 12, 19, 20, 22, 27, 29, 32, 40, 42, 44, 45, 47, 48, 53, 55, 57, 64, 67, 69, 72, 79, 80, 86, 94, 97
