# Supplementary material for: Same old song and dance: an exploratory study of portrayal of physical activity in television programmes aimed at young adolescents
Source: BMC Res Notes. 2018 Jul 11;11:458. doi: 10.1186/s13104-018-3554-8 (PMC6042342; doi:10.1186/s13104-018-3554-8)
Supplement: Supplementary file 2 — Additional file 2. Data Recording Form. [file 13104_2018_3554_MOESM2_ESM.docx]

**Additional file 2: Data Recording Form**

Programme: ____________________ Episode: ___________

| **CUE CHARACTERISTICS** | **Occurrence 1** | **Duration (s) 1** | **Occurrence 2** | **Duration (s) 2** |
| --- | --- | --- | --- | --- |
| **TYPE** |  |  |  |  |
| Visual |  |  |  |  |
| Verbal |  |  |  |  |
| Visual+ verbal |  |  |  |  |
| **FOCUS** |  |  |  |  |
| Main event |  |  |  |  |
| Foreground |  |  |  |  |
| Background |  |  |  |  |
| **CONTEXT** |  |  |  |  |
| Team formal |  |  |  |  |
| Team informal |  |  |  |  |
| Individual formal |  |  |  |  |
| Individual informal |  |  |  |  |
| **SEX** |  |  |  |  |
| Male |  |  |  |  |
| Female |  |  |  |  |
| Mixed |  |  |  |  |
| Major character |  |  |  |  |
| **AGE** |  |  |  |  |
| Child |  |  |  |  |
| Teen |  |  |  |  |
| Adult |  |  |  |  |
| Mixed |  |  |  |  |
| **INTENSITY** |  |  |  |  |
| Mild |  |  |  |  |
| Moderate |  |  |  |  |
| Vigorous |  |  |  |  |
| **DOMAIN** |  |  |  |  |
| Occupation |  |  |  |  |
| Domestic |  |  |  |  |
| Recreational |  |  |  |  |
| Active travel |  |  |  |  |
| **PHYSICAL ACTIVITY** |  |  |  |  |
| Walking (slow) |  |  |  |  |
| Walking (brisk) |  |  |  |  |
| House cleaning |  |  |  |  |
| Occupational |  |  |  |  |
| Playing games |  |  |  |  |
| Soccer |  |  |  |  |
| American football |  |  |  |  |
| Rugby |  |  |  |  |
| Basketball |  |  |  |  |
| Tennis |  |  |  |  |
| Golf |  |  |  |  |
| Dancing |  |  |  |  |
| Cycling |  |  |  |  |
| Skiing |  |  |  |  |
| Skateboarding |  |  |  |  |
| Boxing |  |  |  |  |
| Gymnastics |  |  |  |  |
| Yoga |  |  |  |  |
| Weight lifting |  |  |  |  |
| Jogging |  |  |  |  |
| Swimming |  |  |  |  |
| Running |  |  |  |  |
| Fighting |  |  |  |  |
| Surfing |  |  |  |  |
| Working out |  |  |  |  |
| Other |  |  |  |  |
| **MOTIVATION** |  |  |  |  |
| Be healthy |  |  |  |  |
| Gain fitness |  |  |  |  |
| Enjoyment |  |  |  |  |
| To gain muscle |  |  |  |  |
| To lose weight |  |  |  |  |
| Social |  |  |  |  |
| School curriculum |  |  |  |  |
| Functional |  |  |  |  |
| De-stress |  |  |  |  |
| **SENTIMENT** |  |  |  |  |
| Positive |  |  |  |  |
| Negative |  |  |  |  |
| Neutral |  |  |  |  |

This table was used to record occurrences of physical activity in each programme. Additional columns were added of extra occurrence.

* (s) = seconds
